# Hypoxia signaling pathway: A central mediator in endocrine tumors

**DOI:** 10.3389/fendo.2022.1103075

**Published:** 2023-01-09

**Authors:** Deepika Watts, Mangesh T. Jaykar, Nicole Bechmann, Ben Wielockx

**Affiliations:** Institute of Clinical Chemistry and Laboratory Medicine, Technische Universität Dresden, Dresden, Germany

**Keywords:** hypoxia, endocrinology, tumor, pheochromocytoma, therapy

## Abstract

Adequate oxygen levels are essential for the functioning and maintenance of biological processes in virtually every cell, albeit based on specific need. Thus, any change in oxygen pressure leads to modulated activation of the hypoxia pathway, which affects numerous physiological and pathological processes, including hematopoiesis, inflammation, and tumor development. The Hypoxia Inducible Factors (HIFs) are essential transcription factors and the driving force of the hypoxia pathway; whereas, their inhibitors, HIF prolyl hydroxylase domain (PHDs) proteins are the true oxygen sensors that critically regulate this response. Recently, we and others have described the central role of the PHD/HIF axis in various compartments of the adrenal gland and its potential influence in associated tumors, including pheochromocytomas and paragangliomas. Here, we provide an overview of the most recent findings on the hypoxia signaling pathway *in vivo*, including its role in the endocrine system, especially in adrenal tumors.

## Hypoxia inducible factors: Overview of structure, function, and regulation

Oxygen is essential for the functioning and survival of cells and tissues because it is required for cellular energy production and as a cofactor/substrate for various enzymes; thus, the absence of sufficient oxygen pressure results in hypoxia. However, the term hypoxia is relative and should be used in the correct context as normal oxygen levels vary among tissues, e.g., from ~13% in arterial blood to ~4% in the brain (1). At the molecular level, mechanisms of cellular adaptation to hypoxia involve the hypoxia-inducible factors (HIF-1α, HIF-2α and HIF-3α), which are transcription factors that regulate the expression of hundreds of genes involved in adaptation processes and cell survival. ChIP-seq analysis and genome-wide chromatin immunoprecipitation, combined with DNA microarrays (ChIP-on-chip), have revealed that more than 800 genes regulated by HIFs are involved in various biological functions (2, 3). Furthermore, HIFs also regulate many microRNAs (4) and chromatin-modifying enzymes (5).

**Figure 1:**
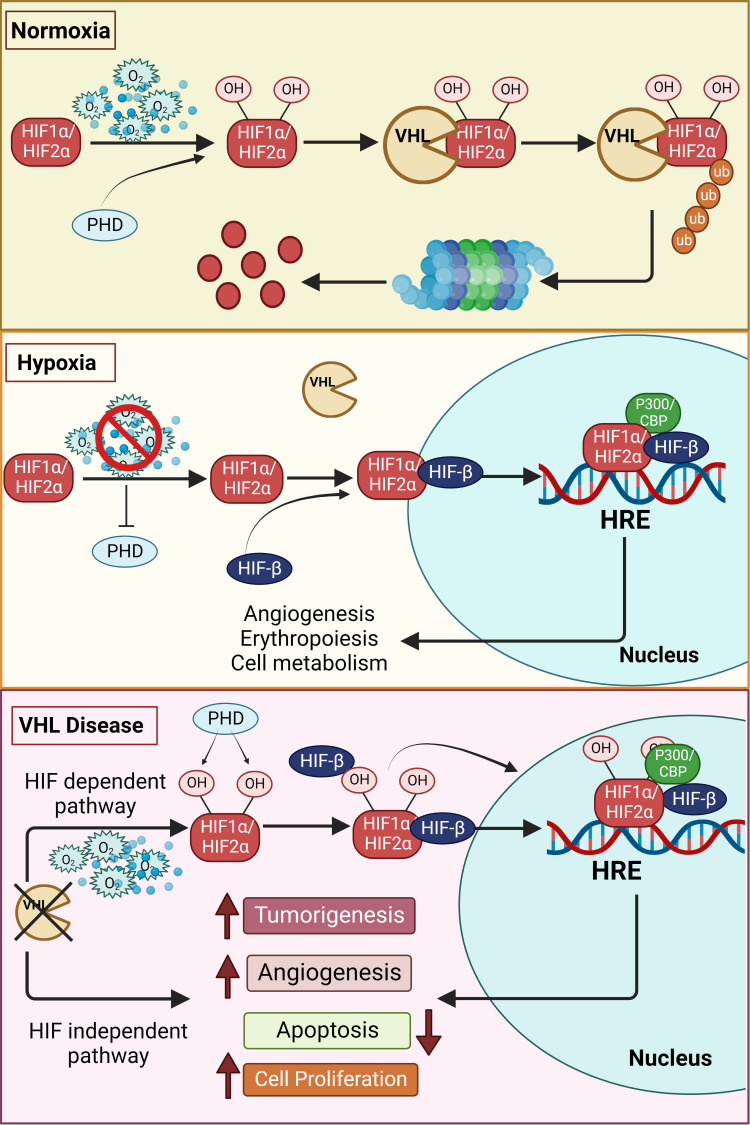
Schematic overview of the hypoxia pathway in normoxia, hypoxia and VHL-disease. In the presence of oxygen, the hypoxia-inducible factors (HIF)-α subunits are synthesized, hydroxylated by HIF prolyl hydroxylase domain (PHDs) proteins in the presence of oxygen, leading to the subsequent binding to von Hippel–Lindau (VHL) tumor suppressor protein. VHL mediated ubiquitination of hydroxylated HIFs, results in proteasomal degradation under normoxia. However, the hydroxylation of the alpha subunits is repressed under hypoxia due to inactivation of PHDs, thus stabilizing the HIF-α subunits. The stabilized alpha subunits bind to the beta subunit and translocate to the nucleus. The HIFs, together with other cofactors, bind to the hypoxia response elements (HREs), promoting the transcription of genes essentially involved in erythropoiesis, angiogenesis and other genes regulated by hypoxia pathway proteins. However, in the VHL disease, even in the presence of oxygen, HIFs are stabilized due to inactivity of the VHL resulting in increased tumorigenesis. This occurs directly by upregulation of tumor-associated genes or is facilitated by suppression of apoptosis and increase in angiogenesis. VHL mediated tumor development can also occur in an HIF-independent manner by upregulation of tumorigenesis. Additional information can be found in the text.

Structurally, each functional HIF transcription factor is a heterodimer of two subunits, an oxygen- sensitive HIF-α subunit and a constitutively expressed HIF-1β subunit, and both these subunits belong to the basic helix–loop-helix (HLH)-PER-ARNT-SIM (bHLH-PAS) protein family (6, 7). The β subunit is also called the aryl hydrocarbon receptor nuclear translocator, ARNT. In the presence of adequate oxygen, HIF prolyl hydroxylases (PHDs) hydroxylate the HIF-α subunit at conserved proline residues located in its oxygen-dependent degradation domain (8, 9). Subsequently, an E3 ubiquitin ligase, von Hippel Lindau (VHL), binds to the hydroxylated HIF-α, leading to its ubiquitination and proteasomal degradation (**Figure 1**) (10, 11). The PHD enzymes belong to the 2-oxoglutarate-dependent oxygenase superfamily and are dependent on oxygen, iron, and ascorbate for their activity (12); hence, in hypoxic cells, neither HIF-1α nor HIF-2α is hydroxylated by the PHDs, which halts HIFs degradation. This results in the accumulation of HIF-1α or HIF-2α in the cell and their consequent binding to the HIF-1β subunit, leading to the formation of the functional HIF heterodimer that then translocates to the nucleus along with its co-activators (p300 and CBP) to form the transcriptional complex. Specifically, the HIF transcription factor binds to hypoxia-responsive elements (A/GCGTG consensus motif) in the promoter region of several genes that regulate various processes such as erythropoiesis, angiogenesis, metabolism, apoptosis, cellular differentiation, and metastasis (**Figure 1**) (13–15).

Another oxygen-dependent mechanism of HIF regulation involves Factor inhibiting HIF-1 (FIH1), an asparaginyl hydroxylase, which hydroxylates the HIF-α subunit at the asparagine in the C-terminal activation domain (N-803 in human HIF-1α) under normoxia and mild hypoxia. Such hydroxylation prevents activation of HIF as it inhibits interactions between HIF-1α and its co-activators p300/CBP (16). FIH1 also acts as a safety net because it is less sensitive to a reduction in oxygen pressure compared to PHDs and remains active even under mild hypoxia to block HIF-α activation that has escaped PHD-mediated degradation (17).

While the above pertained to oxygen-dependent regulation of HIFs, various tumor suppressor and oncogenic pathways (such as MAPK/ERK and PI3K/AKT) non-specifically regulate HIFs in an oxygen-independent manner. Tumor suppressors such as p53 and GSK3β decrease HIF-1α stability or transcriptional activity and thereby interfere with HIF function (18); in contrast, PI3K/AKT pathway activation has been shown to increase HIF-1α mRNA translation and production (19, 20). Another important mechanism of regulation is phosphorylation, which affects both HIF-1α stability and its transcriptional activity. Here, the tumor suppressor GSK3β phosphorylates HIF-1α at three serine residues within the human HIF-1α N-terminal transactivation domain (21, 22), which results in the Fbw7 and USP28-mediated HIF-1α ubiquitination and VHL-independent proteasomal degradation (23). Similarly, HIF-1α is also destabilized by PLK3, which phosphorylates two serine residues (24). Contrarily, HIF-1α phosphorylation can also lead to stabilization because it has been shown that HIF-1α stability increases upon phosphorylation at Ser-696 by ataxia-telangiectasia mutated (ATM) protein kinase (25). Further, ERK1 phosphorylates HIF-1α in its C-terminal activation domain, increasing its transcriptional activity, but not its stability (26).

Notably, even though both HIF-1α and HIF-2α contain similar binding and dimerization domains, they have different transactivation domains (27). Therefore, despite the presence of several common target genes, they can also individually modulate a unique sets of genes (28). Interestingly, these differentially regulated unique target genes can have opposing effects, as recently demonstrated in endothelial cells (29).

### Hypoxia and HIFs in cancers and tumors

Solid tumors are characterized by hypoxic or even anoxic regions within the tumor mass because existing blood vessels fail to meet the oxygen requirements of the rapidly proliferating cancer cells (30). The consequent up-regulation of angiogenic factors, i.e., in response to tumor hypoxia, leads to the formation of non-functional blood vessels with structural and functional abnormalities (31) and this aberrant tumor vasculature severely restrains oxygen supply in the tumor microenvironment resulting in acute hypoxia (32). The involvement of tumor hypoxia in chemo- and radio-resistance is well established (33, 34), and new data suggests its involvement in resistance to immunotherapy as well (35). As the cellular response to hypoxia is controlled by HIF transcription factors that regulate several genes related to adaptation and progression of cancer cells, tumor hypoxia and HIFs govern many attributes of cancer cells, such as proliferation, metabolism, apoptosis, genomic instabilities, vascularization, immune responses, and invasion and metastasis. Typically, sustained hypoxia activates cell apoptosis; however, in tumors, it directs the selection of tumor cells that are resistant to apoptosis, and thereby, contributes to the malignant phenotype (36–38). Understandably, anticancer agents that target rapidly dividing cells are less effective against hypoxic cells that are more distant from vasculature and have reduced rates of proliferation. Moreover, cancer stem-like cells (CSCs) are a rare population of tumor cells that have self-renewal capacity and contribute to treatment resistance. As CSCs reside in the more hypoxic niches of the tumor, they escape chemo- and radiotherapy-induced DNA damage and thereby not only survive treatment but also repopulate the tumor with their progeny (39).

### Hypoxia signaling pathway in endocrine tumors

Endocrine tumors can originate in any of the hormone-producing endocrine organs— thyroid, pituitary glands, pancreas, and adrenal gland, and their nomenclature reflects both origin and location. Thyroid cancer is considered the most common malignancy among all endocrine tumors and anaplastic thyroid carcinomas (ATCs) are predominantly aggressive tumors with an average survival of 3–4 months (40, 41). On the other hand, pheochromocytoma (PCC) and non-secretory pancreatic islet cell cancers are caused by mutations in the VHL tumor suppressor and are characterized by marked interfamilial variations in frequency, significant morbidity and, sometimes, even mortality.

Processes and factors such as signaling pathways, metabolic reprogramming, extracellular matrix remodeling, and epigenetic changes regulate the development and propagation of endocrine tumors (42, 43). As endocrine cancers, like most solid tumors, frequently exhibit major hypoxic areas, hypoxia signaling pathway genes have been associated with endocrine tumor development as well (14, 44–47). Further, given that the hypoxia pathway is one of the major drivers of endocrine tumors, identifying the exact molecular mechanisms of these dysregulated processes could help in the discovery of key therapeutic targets (**Figure 1**).

### Pheochromocytoma and paraganglioma

Pheochromocytomas (PCCs) and paragangliomas (PGLs; together termed as PPGLs) are unique neuroendocrine tumors that make up less than 1% of all endocrine neoplasia. PCCs arise from chromaffin cells of the adrenal medulla whereas PGLs are extra-adrenal, neural crest-derived, neuroendocrine tumors (NETs) of the sympathetic and parasympathetic ganglia (48, 49). PGLs arising from parasympathetic paraganglia are mostly found in the head and neck region, including in the carotid body, due to the presence of neuroendocrine chief cells in the vagus and the glossopharyngeal nerves (50). PCCs and PGLs of sympathetic origin often secrete catecholamines, leading to systemic cardiometabolic effects (51) such as palpitations, tachycardia, hypertension, headaches, diaphoresis, heat intolerance, and anxiety. Even though PCCs and PGLs are typically benign, malignancy can occur in 10–15% of the cases with metastasis to bone, liver, lungs, and lymph nodes (52).

#### Mutations associated with PCCs and PGLs

Approximately 35–40% of PCCs and PGLs have a hereditary predisposition that is attributable to germline pathogenic variants (PVs) in over twenty susceptibility genes (53–55). Rates of predisposition to such germline PVs range between 25–30% in PCC, up to 40% in PGL, and about 50% in metastatic disease (56). Tumors with germline PVs are broadly categorized in two clusters, viz., Cluster 1 (pseudohypoxia) and Cluster 2 (kinase signaling). Cluster 1 includes PV mutations in *SDH*, *VHL*, fumarate hydratase *(FH)* and *EPAS1*, whereas cluster 2 PPGLs bears mutations in *NF1*, rearranged during transfection (RET) proto-oncogene, *TMEM127*, and *MAX*. While cluster 1 PPGLs are characterized by an immature catecholamine phenotype (noradrenergic phenotype) and higher aggressiveness, cluster 2 PPGLs have more mature phenotype (adrenergic phenotype) and are mostly non-metastatic (57, 58). However, in recent years, mRNA expression analysis for The Cancer Genome Atlas (TCGA) has found an additional cluster for PCC/PGL, namely, the *WNT*-altered cluster 3, which is associated with increased expression of genes in the *WNT* signaling pathway (58). Many WNT tumors are driven by novel somatic alterations in *CSDE1* (Cold Shock Domain Containing E1) and recurrent fusions involving *MAML3* and a cortical admixture subtype (58). *CSDE1* is a tumor suppressor gene that encodes the *CSDE1* factor, which is involved in development, messenger RNA stability, internal initiation of translation, cell-type-specificapoptosis, and neuronal differentiation (59). It has been found that the *CSDE1* is significantly mutated in PCCs/PGLs and that these mutations result in the downregulation of the apoptosis protease activator protein 1 (*APAF1*), which is required for controlling apoptosis in PCC cells (60, 61).

Germline and somatic mutations in major susceptibility genes associated with hypoxia signaling involved in the PCC development, include the tumor suppressors such as *VHL1*, the SDH complex (genes encoding the four subunits, A,B,C,D) and occasionally, the egl-9 family hypoxia-inducible factor 1/Prolyl hydroxylase domain 2 protein (*EGLN1/*PHD2) (62, 63). Recently, ten new genes have been added to this list and those associated with the hypoxia pathway include HIF-2α (endothelial PAS domain containing protein 1, *EPAS1*) (64, 65), FH (66), and PHD1 (egl nine homolog 2, *EGLN2*) (67), suggesting that a mutation in any of these major genes involved in the VHL-HIF axis can lead to PCC or PGL development.

#### Von Hippel-Lindau disease

Von Hippel-Lindau (*VHL*) disease is an autosomal dominant neoplastic disorder that is characterized by multiple benign and malignant tumors, including cysts, that develop in the central nervous system and visceral organs (68). Various mutations in *VHL* have been found to cause diverse clinical symptoms; sometimes even the same mutation yields different phenotypes (69, 70). As pVHL has multiple functional domains, one of the potential explanations for this phenomenon is that a specific mutation causes a particular dysfunction. Specifically, mutations in the *VHL* gene on chromosome 3 affect the functionality of pVHL, i.e., as pVHL is incapable of recognizing hydroxylated HIFs, their greater stability leads to HIF-mediated transcription of genes and consequent development of VHL disease (**Figure 1**) (71, 72). The role of *VHL* in disease development has been described in detail elsewhere by Hudler and colleagues (71).

PCC is a hallmark of VHL disease and its absence or presence defines phenotypic classification as VHL type - 1 (protein-truncating mutations) or type - 2, which is linked to missense mutations (68). About 20% of patients with *VHL* disease will develop either unilateral or bilateral intra-adrenal PCC; however, the incidence of extra adrenal PGLs is rare and they occur only in type 2 disease with about 5% of the cases showing metastasis (73, 74). Type 2 disease is further divided into 3 subgroups - 2A, 2B, which correspond to low and high-risk clear cell renal cell carcinoma (ccRCC), respectively, and 2C with only PGL. Interestingly, there is a correlation between degree of HIF dysregulation and mutant VHL alleles in types 1, 2A, 2B, and 2C; specifically, highest HIF dysregulation is seen in type 1 while lowest is seen in type 2C (75, 76). Additionally, existing literature points towards Chuvash polycythemia being a type 3 VHL disease (77, 78). Nevertheless, type 2 VHL disease related PCCs/PGLs show overexpression of several genes involved in angiogenesis, glucose metabolism, and cell proliferation, with the hypoxic pathway particularly associated with stabilization of HIF-2α (**Figure 1**), i.e., the main isoform expressed in catecholamine-producing cells (79, 80). As PCC/PGL-linked biochemical testing is correlated with norepinephrine/normetanephrine predominance, as established from plasma levels, this phenotype is caused by the silencing of the norepinephrine-to-epinephrine converting enzyme Phenylethanolamine-N-Methyl-Transferase (*PNMT*) in the adrenal medulla (45, 81–83). Interestingly, this characteristic is similar to the immature phenotype seen in tumors with *VHL* mutations, i.e., a phenomenon that is associated with HIF-2α overexpression and stabilization. HIF-2α accumulation, rather than HIF-1α, is a major phenomenon in VHL tumors and it results in the overexpression of hypoxia-induced angiogenic genes, such as vascular endothelial growth factor (*VEGF*), erythropoietin (*EPO*) and EPO-receptor, cyclin D1 (*CCND1*), and other genes involved in extracellular matrix reorganization that facilitate tumor development (45, 84). Nonetheless, other HIF-independent proteins and pathways can also be dysregulated in VHL-mutated tumors, e.g., the developmental neuronal apoptosis pathway, p53-related networks, and glucose metabolism. Likewise, the HIF-independent defective apoptosis pathway (for example in type 2C VHL disease) cannot induce apoptosis in chromaffin cells due to greater stability of p53 (85), and some of the type 2C mutations interfere with the regulation of transcription factor AP-1 (JUN)-induced apoptosis due to a VHL-mediated reduction in *Jun-B* and *EGLN3*/*PHD3* levels (71, 86, 87).

#### SDH gene mutations

**Figure 2:**
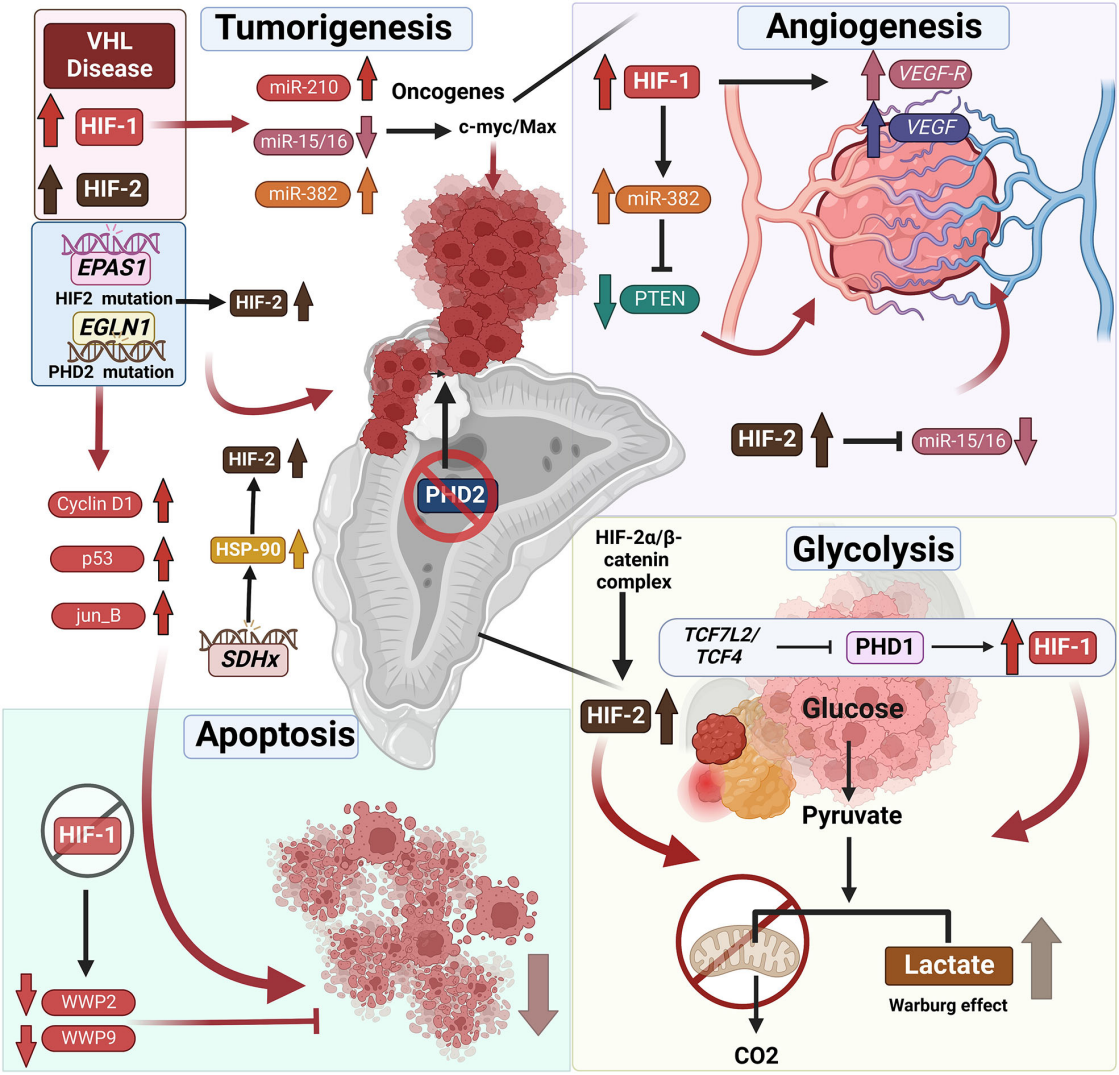
Mutations associated with hypoxia pathway proteins and their role in endocrine tumor development. VHL disease leads to the stabilization of HIFs, which is frequently associated with endocrine tumors – specifically pheochromocytomas (PCCs) and paragangliomas (PGLs). Not only does HIF upregulation result in tumorigenesis directly, it also results in reduced apoptosis of endocrine tumor cells. It was shown that HIF-1α repression induces apoptosis by downregulation of the expression of WW domain containing E3 ubiquitin protein ligase (*WWP9, WWP2)*, promoting tumor cell apoptosis. Furthermore, HIF-1α and HIF-2α stabilization leads to changes in tumor-associated microRNAs. The HIF-associated increase or decrease in micro-RNAs correlated with upregulation of oncogenes and thus tumorigenesis. Likewise, the mutations in the genes such as *EPAS1* or *EGLN1* directly affects the stabilization of the HIFs resulting in tumor development and reduced apoptosis of endocrine tumor cells. *SDHx* mutations in PCCs and PGLs result in the overexpression of the heat shock protein 90 (HSP-90) resulting in the stabilization of HIF-2α. TH : Cre mediated medullar deletion of PHD2/*EGLN1* in the mice resulted in the growth of aberrant structures from the adrenal medulla. Next to the direct effect of HIF stabilization on the tumor development, it also affects angiogenesis by upregulation of *VEGF* and *VEGFR* or miR-15/16 repression initiating angiogenesis. Furthermore, stabilization of HIFs, results in a shift from oxidative phosphorylation to glycolysis, converting pyruvate to lactate in the TCA cycle often mentioned as Warburg effect in the tumors, including endocrine tumors. Additional information can be found in the text.

PCC/PGL development is not only directly associated with mutations in the hypoxia pathway but also correlated to modifications in other genes responsible for HIF stabilization, included in cluster 1. One such example is the succinate dehydrogenase (*SDHx*) family comprising of 4 SDH subunits (A, B, C, D) (47, 88). Mutation in one of the *SDHx* genes promotes accumulation of the oncometabolite succinate, which inhibits the 2-oxogluarate-dependent PHDs and subsequently leading to stabilization and accumulation of HIF-2α (**Figure 2**). Additionally, metastatic SDHx-related PCC/PGL overexpress heat shock protein 90 (HSP90), a molecular chaperone that facilitates binding to HIF-2α by promoting its stability and preventing ubiquitination and proteasomal degradation (89–91).

SDHx-mediated HIF stability is one of the major drivers of PCC/PGL development as it activates genes associated with pseudohypoxia, angiogenesis, protein transport, energy metabolism regulation, and proliferation. Moreover, we and others have previously demonstrated that increased stabilization of HIF-2α is directly associated with a neuroendocrine to mesenchymal transition in these cells, contributing to a more pro-metastatic state of these cluster 1 PPGLs (57, 92). *SDHx*-associated PCCs/PGLs also have a norepinephrine/normetanephrine predominant profile (81) and, like VHL-associated tumors, are associated with *PNMT* gene silencing. Specifically, *SDHB* mutations are predominantly associated with multiple tumors, and although heterozygous *SDHA* pathogenic variants account for less than 1% of all PCC/PGL, *SDHB* mutations account for most common mutation associated with malignant PCCs (93, 94). *SDHA* pathogenic carriers can develop PCC/PGL at any location in the body, including head and neck PGLs and patients who develop *SDHA* mutation related PCC/PGL report to have high rates of metastatic disease (12%) (89, 95). Further, PCC patients with *SDHB* or *SDHD* mutations present overexpression of HIF-2α and its transcriptional target *VEGF*.

#### PHD mutations

The PHD family consists of PHD1, PHD2, and PHD3; and PHD2, encoded by *EGLN1*, is a crucial oxygen sensor that regulates HIFα levels (96). PHD2 dysregulation in the hypoxia pathway results in HIF-2α stabilization and consequent accumulation, leading to a pseudohypoxic state that may underlie the pathologic conditions encountered in PCC/PGL (96). Various mutations in *EGLN1* have been associated with HIF-2 stabilization, e.g., a heterozygous germline mutation at H374 predisposes to instability and loss of PHD2 activity, leading to upregulation of HIF-2α due to greater stability (96). The H374R mutation is also associated with recurrent PGL, suggesting a crucial role for *PHD2/EGLN1* as a tumor suppressor gene that is similar to *VHL* missense mutations seen in type 2 VHL disease, i.e., high risk of PCC/PGL (97). Moreover, Yang and colleagues have demonstrated that a germline mutation in *PHD1* and a novel germline *PHD2* mutation are associated with PCC/PGL and polycythemia (67). Additionally, a unique association between polycythemia and mildly elevated erythropoietin (EPO) levels has been observed in patients, which was linked to inappropriate hypersensitivity of erythroid progenitors to EPO, indicating increased *EPOR* expression/activity (67). Contrastingly, Provenzano and colleagues have recently described a novel germline *EGLN1* gene variant in a patient with metastatic PCC and chronic myeloid leukemia (CML) in the absence of polycythemia (98). Recently, Eckardt and colleagues have reported that inactivation of *PHD2* in the adrenal medulla, using a (Tyrosine Hydroxylase) TH-restricted Cre mouse line, resembles a combination of pseudohypoxic PGL and a PNMT negative noradrenergic phenotype (99). This TH:cre specific deletion of PHD2 was associated with morphological abnormalities in adrenal development, including ectopic TH+ cells (**Figure 2**) (99). Thus, these findings collectively establish that mutations in PHDs are associated with susceptibility to pheochromocytomas and paragangliomas (PPGLs), with or without polycythemia. However, compared to VHL, mutations in PHDs are relatively rare with an incidence of less than 2% in patients with PPGLs (58, 100, 101).

#### HIF stabilization

As mentioned above, upregulation and stabilization of HIFs is widely associated with angiogenesis, tumor progression, and immune evasion in various tumors (46, 102–104), and HIFs are crucial for mediating the tumorigenic effects of mutated *VHL*, *SDHx*, and *EGLN1* in PCC and PGL (105). The role of HIFs in catecholamine synthesis has been extensively studied, and tyrosine hydroxylase (TH), the rate-limiting enzyme in this process that is responsible for the conversion of tyrosine to L-dihydroxyphenylalanine (L-DOPA), can be induced by hypoxia (106). Importantly, both HIFs can bind to the TH promoter and thereby increase TH expression (107). While HIF-2α knockdown has no effect on *Th* mRNA expression in a rodent adrenomedullary chromaffin cell line (108), it seems to be more important for the proper development of differentiated adrenal chromaffin cells. Further, HIF-2α upregulation and accumulation is associated with an immature chromaffin cell phenotype due to reduced PNMT expression and epinephrine synthesis (109). Thus, a noradrenergic phenotype with no epinephrine production is seen upon HIF accumulation during aggressive PGL development (84, 110, 111).

The role of direct mutations in HIFs leading to abnormal stabilization has also been studied in the development of PCC/PGLs, and recently, a transgenic mouse line with whole body HIF-2α gain-of-function mutation showed reduced PNMT in the adrenal glands (64). Moreover, compared to HIF-1α in VHL-related PCC and PGL, HIF-2α stabilization and accumulation is a major phenomenon, and multiple studies have described the oncogenic role of somatic mutations in *EPAS1* in PCC/PGL (105, 112, 113). Consequently, HIF-2α/*EPAS1* has been added to the pool of genes associated with PCC/PGL in the past few years (**Figure 2**) (114). Zhang and colleagues have reported two gain-of-function somatic mutations in exon 12 of HIF2α (c.1588G>A, p.Ala530Thr and c.1589C>T, p.Ala530Val) that result in PGL and polycythemia, respectively (112, 115). Furthermore, two other somatic mutations in HIF2α (c.1595A>G p.Y532C and c.1586T>C p.L529P) in patients with congenital polycythemia, multiple recurrent PPGLs, or somatostatinoma have also been reported (65).

Germline mutations in HIF-2α have also been reported to be associated with PCC/PGL development; nonetheless, as certain germline mutations in HIF-2α only lead to polycythemias and not tumors (115, 116), it is thought that such gain-of-function mutations alone are not sufficient for tumorigenesis, and that, presumably, simultaneous loss-of-function or somatic mutations in other genes may also be necessary (115–117). Another scenario involves gain-of-function mutations in HIF-2α (c.1589C>T) leading to concurrent PPGL and polycythemia (118), as seen in the case of the germline mutation in exon 9 (c.1121T>A, p.F374Y), which predisposes patients to polycythemia and PGL development (119). Further, tumors have been associated with *EPAS1* mutations, either in the absence or presence of polycythemia, and polycythemia alone is seen in cases with mosaic mutations in *EPAS1*. Likewise, Buffet and colleagues have reported the presence of mosaic mutations in two patients with HIF-2α-related polycythemia/PGL syndrome (120). In summary, PHD2 mutations result in a norepinephrine/normetanephrine immature cell phenotype that is caused by silencing of the norepinephrine-to-epinephrine converting enzyme (PNMT) in the chromaffin cells of the adrenal medulla and is related to aggressive PCC development in the presence of mosaic mutations (114, 120, 121).

### Adrenal cortical tumors

Adrenocortical carcinoma (ACC) represent a rare, aggressive, and heterogeneous tumors that arises from the cortex of the adrenal gland. The 5-year survival rate for ACC ranges from 16% to 47% (122). As mentioned above, hypoxia and HIFs are a common feature of many endocrine tumors because these lesions are characterized by rapid proliferation that is associated with metastasis, immune evasion, resistance to therapy, and increased mortality (123). Even though advances over the past few decades in several biomarkers associated with metastasis, prognosis, and survival in ACC patients have led to a better understanding of its molecular genetics, the specific effects of HIF-1 activity in ACC and hypoxia signatures for predicting ACC prognosis have not been established (124). However, a case study of a very rare erythropoietin-producing adrenocortical carcinoma accompanied by lung and liver metastases has been reported (125). Tumor associated hypoxia could be one of the players of erythropoietin production in tumor cells. Recently, a bioinformatic study has reported that a hypoxia-related gene signature could predict prognosis and reflect the immune microenvironment in ACC (126). Specifically, based on hypoxia-related gene expression, ACC patients in the TCGA database were divided into three molecular subtypes (C1, C2, and C3), each with different clinical outcomes with C3 having reported of shortest survival (127).

On the other hand, aldosterone producing adenomas (APAs) are benign aldosterone producing tumors associated with Primary aldosteronism (PA) causing secondary hypertension (128). These are characterized by autonomous production of aldosterone from the adrenal glands leading to low-renin levels and thus hypertension. Most APAs (~ 90%) harbor somatic pathogenic variants in genes encoding ion channels or transporters such as *KCNJ5, ATP1A1, ATP2B3, CACNA1D, CACNA1H, CLCN2*, and *CTNNB1* (128, 129). However, no correlation to hypoxia signaling has been described in the aldosterone producing adenomas until now.

#### Mixed corticomedullary tumor

Mixed corticomedullary tumor (MCT), an extremely rare condition with unclear tumorigenesis, is an adrenal tumor with cortical and medullary cells. Adrenal MCT was first described by Mathison and Water-House in 1969 and only 30 cases have been reported to date. Adrenal tumors, including MCTs, exhibit different stemness expression (130), e.g., a patient with MCT displayed typical Cushing’s syndrome and hypertension, and hence, MCT tumorigenesis is thought to involve the two-hit hypothesis. Pathway enrichment analysis from exosome sequencing of MCT has identified enriched pathways, including the hypoxia-inducible factor-1 (HIF-1) signaling pathway (hsa04066; 1.3%), and some of the germline mutations were involved in stemness regulation, the first hit of which may drive adrenocortical adenoma (ACA) and PCC formation. Additional mutations affecting different pathways, including the HIF-1 signaling pathway, may accelerate tumor growth and intimately mix ACA and PCC (131).

### Additional endocrine tumors

#### Thyroid cancer

As with many types of solid tumors, hypoxia due to inadequate vascularization is a prominent micro-environmental component in thyroid cancer (133). Differentiated thyroid cancer (DTC), which includes papillary cancer (PTC) and follicular cancer (FTC), accounts for >90% of all thyroid cancer cases (132). As angiogenesis is an important factor in the development, growth, and metastasis of cancers, the proangiogenic factor VEGF is a significant mediator of angiogenesis in the thyroid gland. Notably, this makes HIF-1 mediated regulation of VEGF a key factor in the development of thyroid tumors (44, 133, 134). Several studies have reported greater expression of HIF-1α and HIF-2α in thyroid cancer compared to normal thyroid tissue or benign lesions (135–137), and a strong correlation has been observed between HIF-2α expression and tumor size. Specifically, tumors with more intense HIF-1α and HIF-2α staining had a higher TNM stage (137), and overexpression of both HIF-1α and HIF-2α was associated with capsular invasion and lymph node metastasis.

Hyperactive PI3K signaling leads to stabilization of HIF-1α not only in normoxia but also in many cancers, including thyroid cancer and is predominantly involved in the metastasis of PTC and FTC (138, 139). Moreover, Ding and colleagues have reported that silencing of HIF-1α represses cell invasion and induces apoptosis by downregulating the expression of WW domain containing E3 ubiquitin protein ligase (WWP9, WWP2*)*, VEGF, and VEGFR2 in thyroid cancer (44) as depicted in **Figure 2**. Thus, HIF-1α represents a potential therapeutic target for the treatment of thyroid cancer (140).

#### Pancreatic neuroendocrine tumors

Pancreatic cancer is a highly fatal malignancy and the lesion is predominantly characterized by severe hypoxic regions because its median partial oxygen pressure (pO_2_) is 0–5.3 mmHg (0–0.7%); in contrast, the pO_2_ of the adjacent normal pancreatic tissue is 24.3–92.7 mmHg (3.2–12.3%) (141). Thus, pancreatic cancer cells have developed effective adaptive metabolic responses to satisfy oxygen demand for biosynthesis and energy. For example, a major adaptation is the metabolic shift from oxidative phosphorylation to glycolysis, i.e., converting pyruvate to lactate instead of oxidation through the tricarboxylic acid (TCA) cycle (142). More significantly, 5–10% of VHL patients develop pancreatic tumors, which are most commonly non-secretory islet cell tumors known as pancreatic neuro-endocrine tumors (pNET) (143). Patients with missense mutations (type 2 VHL) exhibit a higher prevalence of pancreatic tumors and a hotspot on codons 161/67 in exon 3 is associated with higher risk of metastases compared to truncating mutations or large deletions (type 1 VHL) (144).

In addition to the above, Wnt signaling plays a crucial role in pancreatic tumor development and also alters cell metabolic plasticity to support immediate requirements (145). The transcription factor 7-like2/transcription factor 4 (*TCF7L2/TCF4*) plays a vital role in the Wnt/β-catenin signaling pathway, is responsible for PHD1 (*EGLN2*) silencing, leads to upregulation of HIF-1α, and affects glycolysis reprogramming (146). There is positive-feedback regulation between HIF-2α and β-catenin in that the HIF-2α/β-catenin complex can not only upregulate the activity of β-catenin but also stabilize and increase transcriptional activity of HIF-2α, which then promotes the metabolic shift to aerobic glycolysis in PC cells (147). VEGF stimulation is also known to promote angiogenesis and it can enhance glycolysis in pancreatic cancer by upregulating HIF-1α (148). Targeting the hypoxic tumor environment and the hypoxia pathway in pancreatic tumors has been described in detail elsewhere by Tao et al. (142) (**Figure 2**).

### Micro-RNAs/HIF axis in endocrine tumors

MicroRNAs (MiR) are noncoding, about 20–22 nucleotides in length, and regulate gene expression by binding to 3′ UTRs of their related parental mRNAs. MiRNAs have been shown to control many physiological and pathophysiological processes, such as proliferation, differentiation, metabolism, and apoptosis by modulating target gene expression. Altered miRNA expression has been identified in several endocrine diseases, including tumors. For example, the 14q32 miRNA cluster is frequently dysregulated in human diseases and has been implicated in tumorigenesis of multiple endocrine glands (149). These 14q32 miRNAs may be oncogenic or tumor suppressing depending on cell type and are associated with downregulation of almost all miRNAs encoded by the 14q32 cluster in PCC (149). miR-382, a 14q32.2 miRNA cluster member, is upregulated in PPGLs associated with VHL and SDHB (150), is reported to be an angiogenic miRNA that is upregulated by HIF-1α, and acts as an angiogenic oncogene by repressing PTEN (151).

Several miRNA expression profiling studies have been performed on different endocrine tumors including PCC/PGL, and repression of 2 miRs, namely, miR-15a and miR-16, has been reported in pituitary adenomas and prostate cancer (152). Furthermore, several groups have also studied miRNA expression signatures in benign and metastatic PCC and have reported that miR-15a and miR-16 are indeed under-expressed, and that miR-483-5p, miR-183, and miR-101 are overexpressed in malignant PCC (152). For instance, pre-miR-15a and pre-miR-16 induce cell death and inhibit proliferation in rat PC12 cells but under-expression of miR-15a and miR-16 is associated with malignant tumors rather than benign PGLs. Thus, these miRNAs represent diagnostic and prognostic markers for malignant PCC (152). HIF-2α-induced repression of miR-15 and miR-16 enhances the stability of the c-Myc/Max heterodimer and thereby enhances tumor angiogenesis and metastasis (153). Dysregulation of miR-193b/365 (on chromosome 16p13.12) and miR-183/96 (on chromosome 7q32.2) has been associated with all PCC/PGL and *SDHB*-mutated tumors, respectively (150). Increased miR-21-3p is associated with a general increase in the expression of mesenchymal markers, whereas miR-183-5p decreases the expression of neuroendocrine genes (154). Moreover, compared to benign tumors, miR-101 expression is higher in *SDHD*-mutant malignant PCC tissues (155), and miR-375 is emerging as a new epigenetic alteration that is involved in neuroendocrine tumorigenesis because its overexpression in MTC has been demonstrated both by Hudson et al., and Manso et al. (156, 157).

MiR-210, a recent discovery in the field of hypoxia, is the most consistently and predominantly upregulated miRNA in response to hypoxia. Functional studies have demonstrated that miR-210 is a central gene that regulates many aspects of the hypoxia pathway, both under physiological and malignant conditions (158). MiR-210 is upregulated during hypoxia by HIF-1α (159); hence, it may promote tumorigenesis by activating important oncogenic genes linked to the hypoxia pathway (**Figure 2**) (159). Additionally, Tsang and colleagues have observed that miR-210 is overexpressed in PPGLs associated with *VHL* and *SDHB* germline mutations (159). However, miR-210 overexpression in head and neck PGLs has been reported to be independent of *SDHx* germline mutations, indicating that the HIF-1α/miR-210/*SDHB* axis may play a role in the pathogenesis of PGLs (160). Indeed, further studies are required to better understand the functional interplay between HIFα and miR-210, and its significance in the pathogenesis of PCC/PGLs (**Figure 2**). The role of microRNAs as potential biomarkers and therapeutic targets in PCC/PGL has been described elsewhere by Turai and colleagues (161) (**Figure 2**).

### HIFs inhibitors in cancers/endocrine tumors

Targeting tumor hypoxia and HIFs is an extremely interesting therapeutic approach as tumor hypoxia mediates the aggressive, the metastatic, and the resistant phenotypes. Different approaches have been developed to target hypoxic cancer cells, such as gene therapy, hypoxia-activated prodrugs, recombinant anaerobic bacteria, pathways important in hypoxic cells like mTOR and UPR, and specific targeting of HIFs (162–165). However, HIF targeting is extremely difficult due to the complexity of the HIF pathway and the interconnected signaling cascades involved. Therefore, HIF transcription factors were considered un-druggable; nevertheless, recently, two HIF-2α-inhibitors, PT2385 and PT2399 have been successfully discovered based on the structure of HIF-2α (166–168). These compounds inhibit the growth of ccRCC, both *in vitro* and *in vivo* (169) and a phase I clinical trial yielded complete response, partial response, and stable disease in 2%, 12%, and 52% of the patients, respectively (170).

HIF-2α inhibitors are thought to possess great prospects for the treatment of advanced PPGL (171) and these promising initial results could potentially lead to preclinical and clinical studies to evaluate their efficiency in other types of tumors. Indeed, compound PT2385 is in Phase II clinical trials to assess its effectiveness in advanced cancers with *VHL* germline mutations. Another inhibitor of HIF-2α, PT2977, was found to have increased potency and better pharmacokinetic profile than PT2385 and PT1977 (172). Previous studies have shown that, compared to HIF-1α, HIF-2α is overexpressed in pseudohypoxic PPGLs (92, 173) and contribute to a more aggressive phenotype in these tumors (174). Thus, HIF-2α inhibitors are potentially more promising compared to HIF-1α inhibitors for the treatment of endocrine tumors, especially PCCs and PGLs.

### Summary

Taken together, this review underlines that the hypoxia pathway proteins are essential contributors in the development and progression of endocrine tumors. Even though several mutations such as *VHL, SDHx, EPAS1 and EGLN1* are associated with endocrine tumor initiation, many of these mutations directly or indirectly lead to stabilization of HIFs and consequently triggering the hypoxia pathway. This contributes to tumor development either by upregulation of oncogenes or genes facilitating tumor progression such as angiogenesis, skewed glycolysis or suppression of apoptosis in tumor cells. Therefore, considering the essential role of hypoxia signaling in endocrine tumors, the development of HIF inhibitors as therapeutic agents would be a potential anti-tumor strategy. Considering the upregulation of HIF-2α in many PCC/PGLs, HIF-2α inhibitors are potentially most promising, although more research is certainly warranted.

## Author contributions

DW and MJ, wrote the manuscript. NB and BW contributed to the discussion and edited the manuscript. All authors contributed to the article and approved the submitted version.
